# Ubiquitous Gait Analysis through Footstep-Induced Floor Vibrations

**DOI:** 10.3390/s24082496

**Published:** 2024-04-13

**Authors:** Yiwen Dong, Hae Young Noh

**Affiliations:** Department of Civil and Environmental Engineering, Stanford University, Stanford, CA 94305, USA; noh@stanford.edu

**Keywords:** gait analysis, spatiotemporal parameter, structural vibration

## Abstract

Quantitative analysis of human gait is critical for the early discovery, progressive tracking, and rehabilitation of neurological and musculoskeletal disorders, such as Parkinson’s disease, stroke, and cerebral palsy. Gait analysis typically involves estimating gait characteristics, such as spatiotemporal gait parameters and gait health indicators (e.g., step time, length, symmetry, and balance). Traditional methods of gait analysis involve the use of cameras, wearables, and force plates but are limited in operational requirements when applied in daily life, such as direct line-of-sight, carrying devices, and dense deployment. This paper introduces a novel approach for gait analysis by passively sensing floor vibrations generated by human footsteps using vibration sensors mounted on the floor surface. Our approach is low-cost, non-intrusive, and perceived as privacy-friendly, making it suitable for continuous gait health monitoring in daily life. Our algorithm estimates various gait parameters that are used as standard metrics in medical practices, including **temporal parameters** (*step time, stride time, stance time, swing time, double-support time, and single-support time*), **spatial parameters** (*step length, width, angle, and stride length*), and extracts **gait health indicators** (*cadence/walking speed, left–right symmetry, gait balance, and initial contact types*). The main challenge we addressed in this paper is the effect of different floor types on the resultant vibrations. We develop floor-adaptive algorithms to extract features that are generalizable to various practical settings, including homes, hospitals, and eldercare facilities. We evaluate our approach through real-world walking experiments with 20 adults with 12,231 labeled gait cycles across concrete and wooden floors. Our results show 90.5% (RMSE 0.08s), 71.3% (RMSE 0.38m), and 92.3% (RMSPE 7.7%) accuracy in estimating temporal, spatial parameters, and gait health indicators, respectively.

## 1. Introduction

Gait analysis is a key component in the diagnosis, progression tracking, and rehabilitation of musculoskeletal or neurological disorders, such as cerebral palsy, Parkinson’s, stroke, and dementia [1,2,3]. It typically involves estimating spatio-temporal gait parameters and extracting health-related indicators, such as step time, length, symmetry, and balance [4]. For example, existing studies have shown that estimating spatiotemporal parameters can lead to treatments that can delay the progression and extend patients’ life expectancy [5,6]. In addition, gait parameters indicate the progress of physical rehabilitation, which enables timely interventions that accelerate the process of recovery [7]. Moreover, balance and symmetry indicators have been shown to be critical for fall risk estimation and mitigation for older adults [8]. Quantitative measurements of gait health can help individuals understand their health status and safety risks, leading to improved life quality.

Traditional gait analysis is conducted in gait clinics through direct observation by medical staff, sensing devices such as force plates, electromyography, and motion capture cameras [3,9,10,11]. These approaches can achieve high accuracy in well-calibrated clinics but are unsuitable for ubiquitous gait analysis in daily life because they are expensive and require long calibration time and professionally trained staff to operate [12]. To enable ubiquitous gait analysis, other studies have developed portable cameras, wearable devices, pressure mats, and radio frequency (RF)-based systems for daily tracking [13,14,15,16,17,18,19]. However, they have raised privacy concerns and operational limitations such as direct line-of-sight, having to carry/charge devices, and dense sensor deployment, preventing them from being widely adopted.

**Figure 1:**
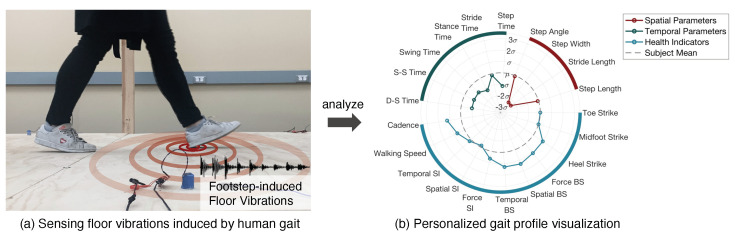
Our approach uses vibration sensors to capture the floor vibrations generated by footsteps during walking (see (**a**)). We develop algorithms to analyze these vibrations, which produce estimates of spatial and temporal gait parameters and gait health indicators. The outcome of our approach is a personalized gait profile (see (**b**)) for individuals to understand their own gait health (colored lines) compared with the average gait from all people (gray dashed circle).

This paper introduces a novel approach for ubiquitous gait analysis using footstep-induced floor vibrations captured in daily living spaces. The main advantages of the floor vibration sensing system include being low-cost, non-intrusive, contactless, and perceived as privacy-friendly when installed in people’s daily living spaces. We use vibration sensors (e.g., geophone and/or accelerometer) to capture the floor vibrations generated by human footsteps during walking (see Figure 1a). The collected data are then analyzed to infer a person’s gait profile in terms of standard gait parameters that are commonly used in medical practices, visualized through a newly designed diagram in Figure 1b. The extracted gait parameters include temporal parameters (i.e., step, stride, stance, swing, double-support, and single-support time) and spatial parameters (i.e., step length, width, angle, and stride length). The extracted gait health indicators include cadence, left–right symmetry, gait balance, and initial contact type, which are important for gait abnormality detection and characterization. While the recent studies have demonstrated promising results in extracting basic gait characteristics from floor vibrations [20,21,22,23,24], their scope is limited and their methods are based on heuristics of the experimental floor. This study closes the gap and introduces a formal framework for ubiquitous and personalized gait analysis.

The main research challenge when developing our method is that the vibration signals are different across various floor structure types [25,26]. This challenge has been highlighted by many previous studies but has never been addressed for gait analysis [25,26]. Specifically, the surface roughness, material properties, and beam/column dimensions and layouts of the floors can vary significantly, making it difficult to develop an algorithm that is generalizable to different floor types. To overcome the challenge, we characterize the vibrations of various floor types and extract features that are insensitive to the floor but sensitive to gait parameters. To this end, our approach can be easily adapted to various building structures, including homes, hospitals, and eldercare facilities.

The core contributions of this paper are as follows:

- We develop a framework for ubiquitous gait analysis using footstep-induced floor vibrations, which is the first of this kind to estimate an extensive range of gait parameters for medical purposes, to the best of our knowledge.
- We characterize the footstep-induced floor vibration to extract features that are sensitive to gait but are adaptable to the changing floor types, improving the scalability of our method for ubiquitous deployment across various floors.
- We evaluate our approach through a real-world experiment with 20 subjects walking on the most common floor types and achieve promising accuracy in gait analysis, with effective visualization using our personalized gait profile diagrams.

To evaluate our approach, we conducted field walking experiments and collected 12,231 gait cycles from 20 subjects on wooden and concrete floors. Our approach has achieved an average of 90.5% (RMSE 0.08s), 71.3% (RMSE 0.38m), and 92.3% (RMSPE 7.7%) accuracy in estimating temporal parameters, spatial parameters, and gait health indicators, respectively. We design personalized gait profiles to visualize a person’s gait overall pattern, allowing observation of gait abnormalities.

In the rest of the paper, we first characterize the floor vibration data (Section 2), present the gait analysis framework (Section 3), and then evaluate our framework through a real-world experiment (Section 4). Finally, we conclude the study and discuss the future work (Section 5 and Section 6).

## 2. Characterization of Footstep-Induced Floor Vibrations for Gait Analysis

In this section, we characterize the floor vibrations induced by gait to understand their relation to spatiotemporal gait parameters and gait health indicators. First, we introduce the physical insight and theoretical basis. Then, we establish the relationship between gait parameters and floor vibration. Finally, we characterize the vibration signals from different floor types to understand the floor effect.

### 2.1. Physical Insight behind Footstep-Induced Floor Vibrations for Gait Analysis

The main physical insight behind floor-vibration-based gait health monitoring is as follows: when walking, a person’s footsteps exert forces onto the floor, which results in dynamic responses of the floor to restore its equilibrium. This footstep-induced repeated deflection–restoration cycle is described as “vibrations” of the floor structure [27,28]. Then, the vibration waves travel through the floor and are captured by the vibration sensors installed on the floor surface at a distance away from the footstep locations. The vibration sensors transform the vertical movements of the floor into electrical voltage time series [29]. Since the variation in the footstep forces leads to distinct floor vibration patterns, we analyze the collected vibration signals to infer human gait characteristics.

The theoretical foundation of the physical relationship between the floor vibration and footstep force is established through the equation of motion in structural dynamics [30], the general form of which can be written as

(1) $$M{\ddot{u}}_{s}\left(t\right)+C{\dot{u}}_{s}\left(t\right)+Ku_{s}\left(t\right)=F\left(t\right)$$

where $u_{s}\left(t\right)$, ${\dot{u}}_{s}\left(t\right)$, and ${\ddot{u}}_{s}\left(t\right)$ are the vertical floor displacement, velocity, and acceleration, respectively, at location *s*; *M* is the floor mass matrix; $C=diag[2\xi_{j}\omega_{j}]$ is the floor damping matrix; *K* is the floor stiffness matrix; and F(t) is the footstep force.

In Equation (1), we observe that the floor vibrations (i.e., $u_{s}\left(t\right)$, ${\dot{u}}_{s}\left(t\right)$, and ${\ddot{u}}_{s}\left(t\right)$) align with the footstep forces F(t) over time and share proportional amplitudes. In addition, the floor vibrations at each location are determined by a unique set of mass, stiffness, and damping parameters, allowing for location-based analysis. This establishes the theoretical foundation of using floor vibrations for temporal and spatial gait analysis.

### 2.2. Relationship between Human Gait and Footstep-Induced Floor Vibrations

In this subsection, we characterize the relationship between human gait and the floor vibration signals. Human gait is typically described by various characteristics during walking in terms of duration, lengths, angles, and forces. The gait characteristics examined in our study include (1) temporal gait parameters (duration measurements), (2) spatial gait parameters (location measurements), and (3) gait health indicators (qualitative measurements such as initial contact type, balance, and symmetry). These are commonly used measurements in clinical gait analysis, which have been shown to be effective and generally applicable for numerous disease types [3,31].

#### 2.2.1. Temporal Gait Parameters

We first introduce the background of temporal parameters in the context of gait cycles and then discuss their relationship with the footstep-induced floor vibrations.

**Background of Gait Cycle and Temporal Parameters.** A gait cycle is the most fundamental concept in human walking, which can be divided into two primary phases: the stance phase and the swing phase. The stance phase is the time when the foot is in contact with the floor; the swing phase is the time when the foot is swinging in the air. The temporal gait parameters are defined based on the duration of stance/swing phases and double/single support time. Estimating the duration of these phases helps to identify potential gait abnormalities. For example, a shorter stance time on one leg indicates asymmetrical gait and difficulty in maintaining balance while walking, which may lead to an increased risk of falls [32].

**Figure 2:**
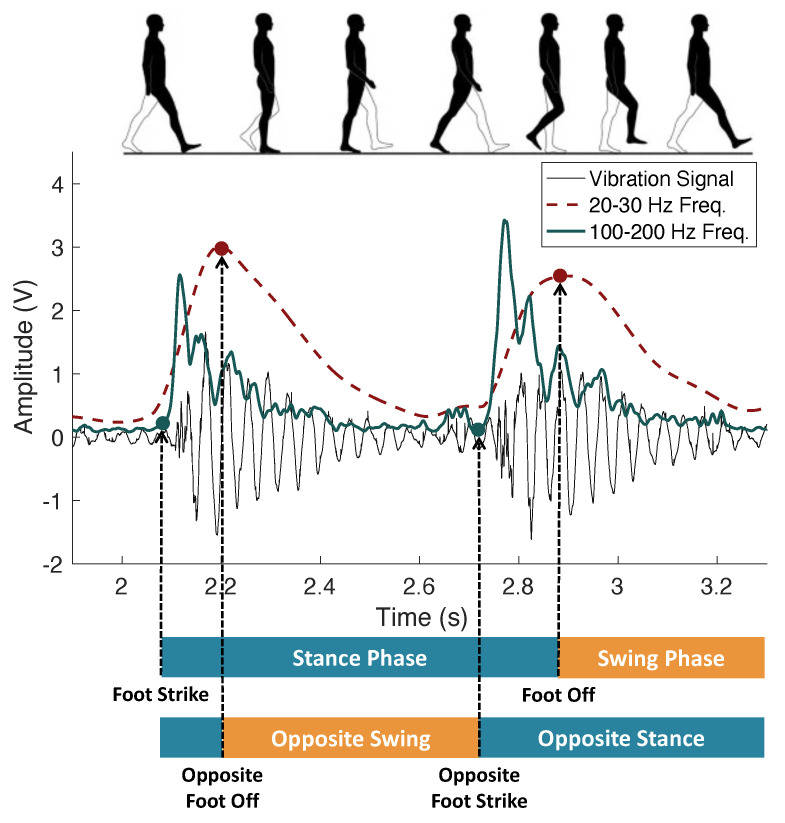
Visualization of temporal gait parameters in relation to footstep–induced floor vibration signals on a wooden floor: a normal foot strike occurs at the beginning of the high–frequency impulses (green solid line), while a normal foot off is at the peaks around the natural frequency of the floor structure (red dashed line).

**Relationship between Temporal Parameters and Floor Vibrations.** To estimate temporal gait parameters from floor vibration signals, we characterize the footstep-induced floor vibration with respect to foot strikes and foot offs. Figure 2 illustrates how various frequency ranges in the floor vibration signals are related to the gait phases.

As observed in Figure 2, the floor vibration induced by a gait cycle consists of two consecutive impulsive signals induced by the left and right foot. This is because the gait cycles from the left and right foot overlap while walking. After the first footstep impulse, the second impulse happens when the person changes the supporting foot, which is marked as the opposite foot strike. In addition, we observed that foot strikes and foot offs occur in their corresponding frequency ranges. Especially, foot strikes typically induce a higher frequency in vibration signals than the foot off because of the impulsive footstep force at the foot strike. On the other hand, foot offs typically occur at the peaks of the low-frequency component (around the natural frequency of the floor) as the body weight shifts forward. This aligns with our intuition that the foot off signifies the beginning of free vibration (i.e., floor vibration when the foot swings in the air), where the natural frequency component reaches the maximum and starts to attenuate. This frequency–gait phase relationship allows us to divide the gait cycle and extract temporal gait parameters, which will be discussed further in Section 3.

#### 2.2.2. Spatial Gait Parameters

Similar to the previous subsection, we first introduce the background of spatial gait parameters and then discuss their relationships with footstep-induced floor vibrations.

**Figure 3:**
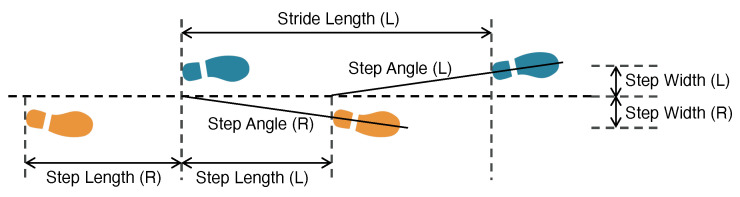
An overview of the spatial gait parameters estimated in this work.

**Background of Spatial Parameters.** The spatial parameters describe the distance and direction of the walking path, as described in Figure 3. When a person is walking in a straight line, the step length is the distance between left and right foot strikes; the stride length is the distance between the two adjacent strikes from the same foot; the step width is the distance between the center of the footstep and the projected footstep center to the walking trajectory; and the step angle is the angle of this projection by setting the previous foot strike location as the origin.

**Relationship between Spatial Parameters and Floor Vibrations.** In order to estimate spatial parameters from floor vibrations, we need to accurately estimate footstep locations because all the spatial parameters in Figure 3 are calculated based on the locations. Previous studies have explored the time-difference-of-arrival (TDoA) method, which provides an around 0.5 m error for footstep localization [33]. However, this error is too large for spatial gait parameter estimation because the average step length of an adult is around 0.5 m [31], meaning that the existing work has a 100% error rate.

The main research barrier that prevents accurate footstep localization using floor vibrations is the heterogeneity of the floor structures, leading to uncertain wave propagation velocities for location estimation. This has long been a challenging problem to address because the wave propagation velocity is typically unknown and is affected by complex factors, including material properties, defects/cracks in the structure, and the properties of the connections between structural components. As a result, when a person’s footstep location changes, the underlying structural property also changes, resulting in a different wave propagation velocity.

**Figure 4:**
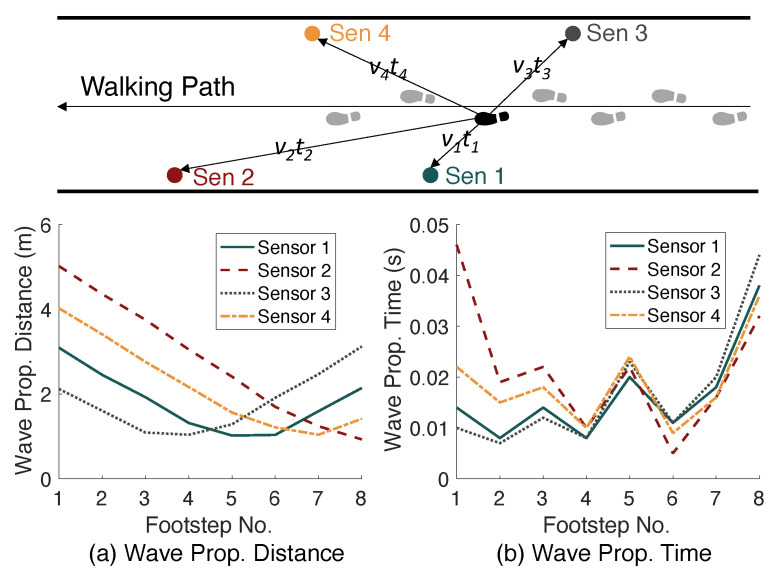
Floor Heterogeneity leads to varying wave propagation velocities at various footstep locations, observed from (**a**) wave propagation distance and (**b**) wave propagation time. Since (**a**,**b**) have different trends, the resultant velocity (distance divided by time) varies among multiple footstep locations.

To understand the effect of floor heterogeneity on vibration wave propagation, we characterize the wave velocity by measuring the wave propagation distance and time when a person walks by. Figure 4 shows the changes in distance and time when the vibration wave propagates from the footstep location to the sensor location. Since the sensors are mounted at different locations, the wave propagation distance reaches the minimum from left to right as the person approaches each sensor, as shown in Figure 4a. However, the wave propagation time (see Figure 4b) follows a different trend where the minimum of each line does not align with the minimum in the distance figure. This means that the velocity varies across different locations on the floor. By taking the division between distance and time (i.e., $v=\frac{\Delta d}{\Delta t}$), we found that the velocity is between 30 m/s and 300 m/s on the testing walkway, which can be the main source of uncertainty in footstep localization.

In addition, the characterization shows that the wave propagation direction also affects the velocity. For example, for the fifth footstep in Figure 4 (highlighted as black in the upper diagram), the wave traveling velocity varies from 70 to 130 m/s among these four sensors. Compared with the velocity variation range among various locations (30–300 m/s), the effect of propagation directions is less significant. Therefore, we assume that the mean velocity of all sensors is representative of the wave traveling velocity at each footstep location as it is not practical to estimate the velocity in a space with an unlimited number of locations and directions. With the above assumption and observations, we calibrate the floor by developing a spatially varying profile of wave propagation velocities, which helps to reduce the uncertainty and improve the localization accuracy. The calibration process will be discussed in Section 2.3.2.

#### 2.2.3. Gait Health Indicators

Gait health indicators are measurements of qualitative health metrics such as balance, symmetry, and foot-floor contact types. In this subsection, we first introduce the background of gait health indicators and then characterize the floor vibration signals induced by various initial contact types.

**Background of Gait Health Indicators.** The gait health indicators estimated in this study include (1) cadence/walking speed, (2) symmetry, (3) balance, and (4) initial contact type. Their definitions and relationships with gait health are discussed in the following paragraphs.

First, cadence is the number of steps taken per minute while walking, and walking speed is the amount of distance traveled at a given time. A slower cadence/walking speed is typically associated with an increased functional decline in walking.

Symmetry refers to the similarity and coordination between the left and right sides of the body while walking [4]. A lack of symmetry can cause an uneven distribution of weight which can increase fall risks and lead to musculoskeletal pain or injury.

Gait balance refers to maintaining stability and control during walking, where a person adjusts postures and movements in response to changes in the walking conditions or external disturbances. To assess gait balance during realistic walking scenarios, we define a balance score based on the variability of the footsteps, introduced in Section 3.4.3.

**Figure 5:**
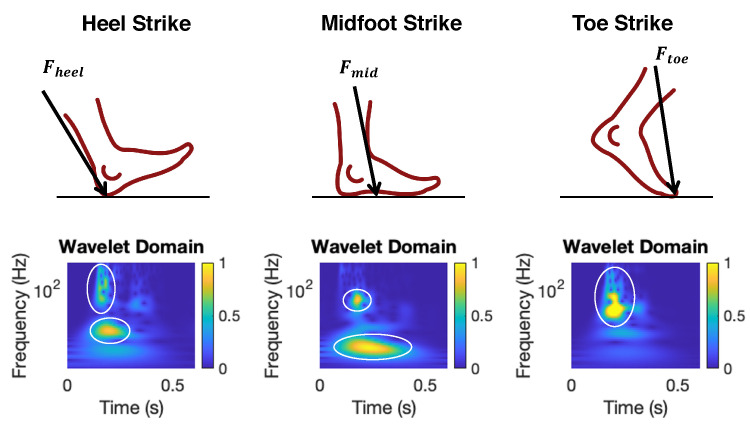
Examples on the wavelet domain of floor vibration signals under three types of initial contacts. The areas within the white circles show distinct dominant frequency patterns across various initial contact types.

Initial contact type refers to the pattern when the foot contacts the floor during the foot strike. There are three main types of initial contact: heel strike, toe strike, and midfoot strike (see Figure 5). The type of initial contact is determined by muscle activation and affects the force transmission through the body during walking, which is important to differentiate disease types and understand disease stages [34,35].

**Relationship between Initial Contact Types and Floor Vibrations.** Each type of initial contact leads to a distinct pattern in floor vibrations, which is shown in the wavelet domain plots in Figure 5. The heel strike induces a higher frequency at the initial contact and a lower frequency during the later progression of the foot. The midfoot strike leads to a lower frequency than that of the heel strike because the footstep force is less impulsive. In contrast, the toe strike results in mainly high-frequency components due to the lack of foot progression on the floor. Therefore, we leverage the wavelet domain features to predict the initial contact types, which will be discussed in Section 3.4.

### 2.3. Effect of Floor Types on Floor-Vibration-Based Gait Analysis

To address the core research challenge of floor type variations, we characterize the vibration signals from various types of floor structures to understand their effect by focusing on temporal and spatial parameters.

#### 2.3.1. Floor Type Influence on Temporal Parameters

To understand the floor type influence on temporal parameters, we first formulate the problem based on theoretical analysis in structural dynamics and then characterize different floor vibrations through controlled experiments.

**Time–Frequency Analysis of the Floor Influence.** Prior work formulated the influence of floor types through the governing equations in structural dynamics by assuming that the floor is a linear time-invariant system [24,26]. The formulation is based on the equation of motion in structural dynamics described in Equation (1). To observe the frequency domain influence of the structure, we apply the Fourier Transform and re-write the expression as follows:

(2) F{u(t)}=H(ω)F{F(t)}⇒Y(ω)=H(ω)X(ω)

where Y(ω) is the frequency spectrum of the floor vibration, X(ω) is the Fourier transform of the footstep force, and the influence of the floor structure is encoded in the transfer function H(ω). Based on modal decomposition, each element $h_{j}\left(x,l\right)$ of the transfer function H(ω) can be written as

(3) $$h_{j}\left(x,l\right)=\phi_{jx}FRF_{j}^{*}\left(\omega_{j}\right)\phi_{jl}$$

where *j* is the mode number and x,l are the given sensor and footstep location; $\phi_{jx}$ and $\phi_{jl}$ are constant values representing mode shapes of a given floor. Since the frequency response function $FRF_{j}^{*}\left(\omega_{j}\right)$ has large values only when $\omega_{j}$ is close to the modal frequency, the footstep force spectrum X(ω) will be amplified through the multiplication of transfer function around the modal frequencies. As a result, the effect of the floor types on temporal parameters is reflected through the dominant frequency components of the vibration signals.

**Figure 6:**
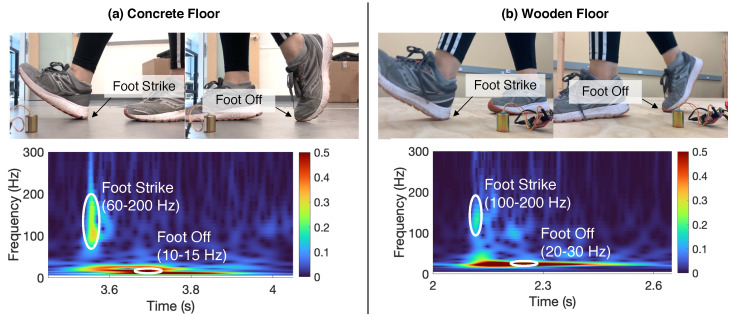
Floor vibrations induced during walking on (**a**) concrete floor and (**b**) wooden floor. The foot strike and foot off are shown in **different frequency ranges** of the time–frequency spectrum after wavelet transform.

**Experimental Observation of Temporal Parameters on Two Floors.** We validate our theoretical analysis through a controlled experiment on a wooden and concrete floor. Figure 6 shows the time–frequency decomposition of the vibration signals of single footsteps. To control the variables, both signals are induced by the same person wearing the same pair of shoes with the same footstep-to-sensor distance. We observe that foot strike and foot off induce different frequency components, which aligns with the theoretical derivation in Equation (3), indicating that the footstep force spectrum is amplified around the modal frequencies at each structure. In addition, we observe that the foot strike/foot off results in different frequencies across these two floors. This is because the wooden and concrete floors are different in mass, stiffness, and damping ratio, leading to discrepancies in dynamic responses. Overall, the foot strike induces a higher frequency at the beginning of the spectrum and the foot off induces a lower frequency at the middle of the spectrum, regardless of the floor type. This allows us to develop floor-adaptive algorithms for temporal parameter estimation.

#### 2.3.2. Floor Type Influence on Spatial Parameters

To explore the floor type influence on spatial gait parameters, we first model the heterogeneity in each floor and then characterize the floor effect based on experimental observations on the two typical floor types.

**Spatial Floor Heterogeneity Modeling.** To overcome the challenge between different floor types, we model the wave propagation velocity profile using several initial trials of walking with a temporarily installed camera to provide ground truth on footstep location and time. To reduce the number of unknowns in the model, we simplify the multi-dimensional floor heterogeneity by assuming that the wave propagation velocity is consistent among various directions. This is because the effect of direction is found to be less significant than the effect of footstep location, as discussed in Section 2.2.2. Under this assumption, we estimate velocity at each footstep location by dividing distance over time. After that, we conduct a non-linear regression on the footstep samples to reduce the effect of outliers and the effect of wave propagation directions. We utilize a fourth-order polynomial regression model for two reasons. First, the cross-section layouts of the testing walkways have two spans, each requiring two orders to fit the parabolic shape of the deformation. Secondly, the polynomial order needs to be constrained to achieve consistent training and validation accuracy without over-fitting individual data samples that reflect local defects. The fitted velocity *v* at location *x* in our case is described as follows:

(4) $$v\left(x\right)=\beta_{4}x^{4}+\beta_{3}x^{3}+\beta_{2}x^{2}+\beta_{1}x+\beta_{0}$$

where $\beta_{i}$ represents the coefficients estimated during the regression.

**Figure 7:**
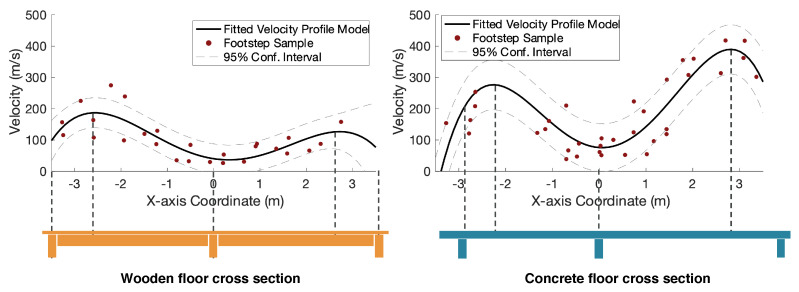
Visualization of velocity profile models for wooden and concrete floors, respectively. Overall, the velocity profile correlates well with the structural layout—the symmetrical wooden floor structure has a symmetrical velocity profile, and the asymmetrical concrete spans are reflected in the asymmetrical velocity profile. The velocity in concrete is generally higher than that in wood.

**Experimental Observation of the Spatial Velocity Profiles on Two Floors.** We validate the floor heterogeneity model through a controlled experiment on a wooden and concrete floor. Figure 7 shows the velocity profile along the longitudinal center line of two types of testing floors. The left is a wooden-framed structure with two spans of the same length. On the right is a concrete floor with two spans of different lengths. The fitted wave velocity curve shows that the concrete floor has significantly higher velocities than the wooden floor, which is consistent with the physical insight that concrete typically has a higher density than wood. In addition, the estimated velocity profile correlates well with its cross-section layout—the vibration wave travels slower at column locations and faster at the mid-span of the structure, which aligns with the fact that the column is typically stiffer than the beams and slabs.

## 3. Ubiquitous Gait Analysis Framework Using Footstep-Induced Floor Vibrations

**Figure 8:**
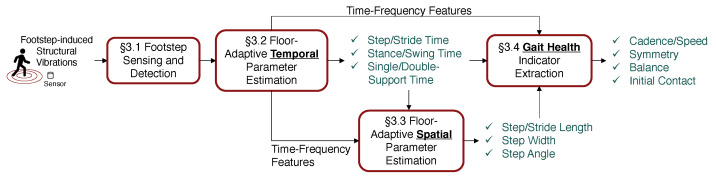
The framework of gait analysis through footstep-induced floor vibrations has four modules (red boxes), including (1) footstep sensing and detection, (2) floor-adaptive temporal parameter estimation, (3) floor-adaptive spatial parameter estimation, and (4) gait health indicator extraction. The data flow pipeline is represented by black solid lines. The outcomes of the framework are highlighted in green-colored text.

In this section, we introduce our ubiquitous gait analysis framework, which estimates spatiotemporal gait parameters and extracts gait health indicators using footstep-induced floor vibrations, and is designed to be robust to various floor types (see Figure 8).

### 3.1. Footstep Sensing and Detection

The footstep sensing and detection module includes three parts: (1) vibration data collection, (2) noise filtering, and (3) individual footstep detection. First, we present the hardware for data collection. Then, we describe the noise filtering process, which aims to handle electrical and environmental noises. After that, we introduce the algorithm that detects individual footstep-induced impulses from the time series data stream.

**Figure 9:**
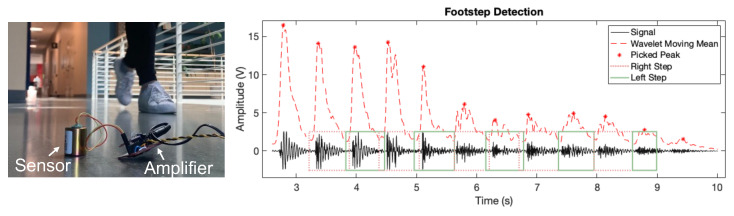
Footstep-induced floor vibration sensing using geophone sensors mounted on the floor surface (**left**). A sample series of detected footsteps through peak-picking of the wavelet coefficients (**right**).

Our sensing system uses floor-mounted geophone sensors to collect footstep-induced floor vibrations, as shown in Figure 9 (left). Geophone sensors are mechanical vibration sensors that convert the velocity of the floor vibrations into an analog voltage signal, which are commercially available at a relatively low cost [29,36]. The sensors are typically connected to operational amplifiers (op-amp) to increase the signal amplitude while choosing the appropriate amplification factors to avoid signal clippings. The amplified analog signals are then converted into digital signals through the Data Acquisition System NI-DAQ [37] from National Instruments at Austin, TX, USA. The effective sensing range after amplification can achieve up to 20 m based on our prior studies [25,38], enabling sparse sensor deployment in daily living spaces.

The noise filtering process typically involves a lowpass filter and a Wiener filter. The lowpass filter is used to remove high-frequency electrical noise. For temporal parameter estimation and health information extraction, the threshold of the lowpass filter is set to 500 Hz to preserve the majority of the effective gait information in floor vibration data (from 5 to 250 Hz) [23]. This threshold is determined by comparing the footstep frequency spectrum and the ambient noise frequency spectrum through preliminary data collection. For spatial gait parameter estimation, the lowpass filter is set to 2500 Hz to compensate for the high wave propagation velocity through the floor medium, enabling an around 10 cm footstep localization resolution through the time-difference-of-arrival (TDoA) method. On the other hand, the Wiener filter is used to reduce environmental noise [39], which takes in 3 s of signal with only the environmental noise and leverages its frequency spectrum to filter out noise on the signal with combined footstep impulses and environmental noises.

The footstep detection algorithm is developed based on peak-picking of the wavelet coefficients. As shown in Figure 9 (right), we conduct wavelet transform of the entire signal using the Morlet wavelet, a commonly used wavelet that is efficient in computation and well-suited for time-varying, non-stationary signals [40]. Since footstep-induced vibration signals are impulsive in nature due to the short foot–floor contact duration, we focus on the natural frequency range of typical floor structures (5–50 Hz) in the wavelet coefficients to detect the peaks where these impulses occur. The peaks are identified based on the empirical observation of pure noise signals when no person passes by, including a (1) minimum amplitude of the peaks, which has to exceed the mean of the pure noise signal plus three standard deviations, and a (2) minimum prominence between adjacent peaks, which has to be larger than three standard deviations of the noise. These adaptive thresholds allow the system to adapt to various noise conditions and amplification settings. In addition, since footsteps typically occur in groups with repeated patterns in the vibration signals as a person walks by, we set the minimum number of continuous impulses to three so that footsteps are distinguished from other human-induced impulse signals such as item dropping and door opening/closing. When detecting the footsteps, two adjacent footsteps are marked from the left and right foot, respectively, to prepare for gait symmetry analysis in Section 3.4.

### 3.2. Floor-Adaptive Temporal Parameter Estimation

The temporal gait parameters we estimate include step time, stride time, stance time, swing time, single-support time, and double-support time. These are critical time durations within a gait cycle.

**Figure 10:**
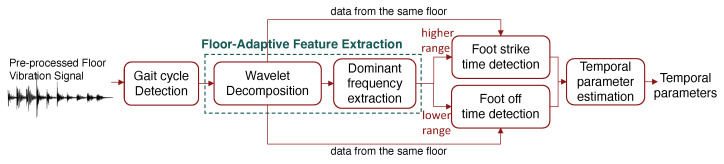
An overview of the temporal gait parameter estimation process.

Our approach for floor-adaptive temporal parameter estimation has four steps: (1) gait cycle segmentation, (2) floor-adaptive feature extraction, (3) foot strike and off time detection, and (4) temporal parameter estimation. Figure 10 shows the estimation process.

First, we detect gait cycles by grouping the previously detected individual footsteps. As introduced in Section 2.2.1, since a typical gait cycle has two foot strikes (including one foot’s strike and the opposite foot’s strike), we combine each pair of consecutive left and right footsteps as a gait cycle group.

Then, we develop floor-adaptive algorithms to extract features from the vibration signals, which are the dominant frequency ranges at each gait event. As discussed in Figure 6 in Section 2.3.1, the main difference between the vibration signals from two different floors is the dominant frequency ranges at the foot strike and foot off. The dominant range for foot strike is typically around 10–30 Hz and that of the foot off is around 60–200 Hz, depending on the type of floor. Therefore, we determine the dominant frequency range by cropping out the first 0–10% of the gait cycle (when foot strike occurs) and 60–70% of the gait cycle (when foot off occurs) to capture the floor difference. Although people’s walking patterns may vary due to individual habits, studies found that the proportion in a gait cycle when the foot strike and foot off occur are relatively consistent [41]. Therefore, the choice of these ranges captures the time when foot strike and foot off happen while allowing flexibility due to person-to-person variability. When a new trace of footsteps is observed from the same floor, we accelerate the process by skipping the dominant frequency extraction step.

Next, we detect foot strikes and off time to remove the effect of the floors. We start off by computing the sum of wavelet coefficients over frequency within the extracted dominant frequency ranges, resulting in two time series. The higher range is for foot strike and the lower range is for foot off based on the floor types characterization in Section 2.3.1. Then, we conduct peak-picking among the resultant wavelet coefficient time series to detect the time for foot strike and foot off. We apply a reverse sliding window starting from the peak to the valley to identify the time when the vibration starts to rise as the foot strike time. On the other hand, the peak of the lower frequency component is determined as the foot off time because it is when damped free vibration starts to attenuate the signal. Finally, each gait cycle is segmented based on the foot-strike and foot off times to compute the temporal gait parameters.

Finally, given the estimated foot strike time $t_{i}^{s}$ and foot off time $t_{i}^{o}$ for the *i*-th gait cycle, as described in Figure 2 in Section 2.2.1, the gait parameters are estimated as follows:

- Step Time = $t_{i+1}^{s}-t_{i}^{s}$;
- Stride Time = $t_{i+2}^{s}-t_{i}^{s}$;
- Stance Time = $t_{i}^{o}-t_{i}^{s}$;
- Swing Time = $t_{i+2}^{s}-t_{i}^{o}$;
- Single-Support Time = $t_{i+1}^{s}-t_{i-1}^{o}$;
- Double-Support Time 1 = $t_{i-1}^{o}-t_{i}^{s}$;
- Double-Support Time 2 = $t_{i}^{o}-t_{i+1}^{s}$.

where $t_{i-1}^{o}$ is the previous gait cycle’s foot off (i.e., opposite foot off) and $t_{i+1}^{s}$ is the next gait cycle’s foot strike (i.e., opposite foot strike). For a given gait cycle, the single support time refers to the opposite swing phase. The first double support time is from the foot strike to the opposite foot off (the initial blue section at the opposite foot bar in Figure 2), and the second double support time is from the opposite foot strike to the current foot off time.

### 3.3. Floor-Adaptive Spatial Parameter Estimation

The spatial gait parameters we estimate include step length, stride length, step width, and step angle. These are estimated based on the footstep location during walking, which is important evidence to assess mobility, symmetry, and balance of walking.

**Figure 11:**
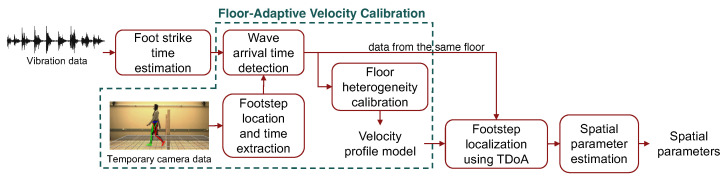
An overview of the spatial gait parameters estimation process.

Our approach for floor-adaptive temporal parameter estimation has four steps: (1) foot strike time estimation, (2) floor-adaptive velocity calibration, (3) footstep localization, and (4) spatial parameter estimation. Figure 11 shows the estimation process.

First, we estimate the time of foot strikes using the extracted dominant frequencies discussed in Section 3.2. This sets a foundation for wave arrival time detection. Then, we calibrate the floor heterogeneity caused by the variations in wave propagation velocity in order to achieve a higher accuracy in spatial parameter estimation, as discussed in Section 2.3.2. The calibration involves setting up a temporary camera that records the step location and time for several walking trials. Through a combined analysis of camera and vibration data, we can estimate the spatial distribution of the wave propagation velocity as developed in prior work [21]. To achieve this, we first estimate the wave arrival time by a peak-picking algorithm on signals between the foot-strike time and the time when the peak occurs in the higher frequency component. This is based on the domain knowledge that the footstep force gradually increases after the initial contact, resulting in a wave arrival time between the foot strike and the peak amplitude. Then, we integrate the results from various sensors to finalize the wave arrival time. Since the sensor closer to the footstep typically receives the wave first, we select the arrival time sequence based on the sequence of footstep-to-sensor distances. With the estimated wave arrival time and wave traveling distance, we model the velocity profile based on Equation (4) in Section 2.3.2 to reduce the influence of outliers and wave propagation directions. The output of the calibration process is a velocity profile model of the cross-sectional area of the floor structure.

It is worth mentioning that our approach remains functional even without the camera-based calibration. In this case, the wave arrival time detection solely relies on the vibration data. Then, instead of estimating the velocity profile through cameras, we determine the wave propagation velocity of each footstep location by minimizing the area of possible footstep locations among three nearby sensors, as introduced in a previous study on vibration-based footstep localization [33]. The resultant wave propagation velocity at each footstep location is used as the velocity profile model.

Next, we leverage the estimated velocity profile model to enhance the localization performance of the TDoA method discussed in Section 2.2.2, where the difference in arrival time across multiple sensors is computed to estimate the footstep location [33]. To achieve this, we first estimate the wave propagation velocity range based on the velocity profile model and the projected footstep location based on previous observations. Then, we compute the TDoA of the wave among various sensors by calculating the relative wave arrival time compared to the anchor sensor (the sensor with the best signal-to-noise ratio). The location of the footstep is predicted through a grid search over the projected footstep range, where the location that leads to the lowest TDoA error is used.

**Figure 12:**
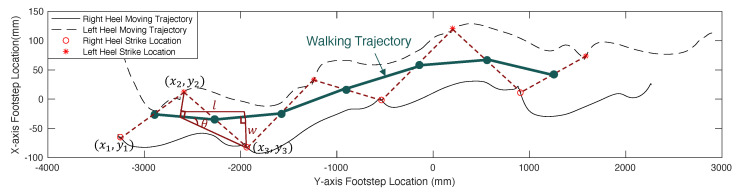
A sample walking trajectory estimated from the heel strike locations.

Finally, the spatial gait parameters are computed according to Figure 3 in Section 2.2.2. Since the walking trajectory of an individual may not be perfectly straight, we estimate the walking trajectory for every three footsteps through a linear regression over the center points of the adjacent footstep locations. Given footsteps $\left(x_{1},y_{1}\right)$, $\left(x_{2},y_{2}\right)$, and $\left(x_{3},y_{3}\right)$ described in Figure 12, the first walking trajectory segment is estimated as

(5) $$y=k_{1}x+b_{1}\;\Rightarrow \;k_{1}=\frac{y_{1}-y_{3}}{x_{1}-x_{3}},\;b_{1}=\frac{y_{1}+y_{2}}{2}-\frac{x_{1}+x_{2}}{2}\frac{y_{1}-y_{3}}{x_{1}-x_{3}}$$

where $k_{1}$ and $b_{1}$ describes gradient and interceptions for the 1st walking trajectory segment.

After repeating the calculation for all the walking trajectory segments, we form a complete walking trajectory (marked as a thick green line in Figure 12). Then, we project each individual footstep to the walking trajectory (see the projected walking trajectory for $\left(x_{2},y_{2}\right)$ and $\left(x_{3},y_{3}\right)$ in Figure 12). Take the 3rd footstep $\left(x_{3},y_{3}\right)$ as an example; the projection distance *w* is computed as the step width, and the distance between projected points *l* is computed as the step length. Based on trigonometry, the detailed calculation is summarized below:

- Step Length: $\begin{array}{c} l_{i}=∥\left(\frac{x_{i-1}+x_{i}}{2},\frac{y_{i-1}+y_{i}}{2}\right)+t_{i}\left(\frac{x_{i+1}-x_{i-1}}{2},\frac{y_{i+1}-y_{i-1}}{2}\right)\\ -[\left(\frac{x_{i}+x_{i+1}}{2},\frac{y_{i}+y_{i+1}}{2}\right)+t_{i+1}\left(\frac{x_{i+2}-x_{i}}{2},\frac{y_{i+2}-y_{i}}{2}\right)]∥\\ \mathrm{where}\;t_{i}=\frac{\left(x_{i}-x_{i-1}\right)\left(x_{i+1}-x_{i-1}\right)+\left(y_{i}-y_{i-1}\right)\left(y_{i+1}-y_{i-1}\right)}{{\left(x_{i-1}-x_{i+1}\right)}^{2}+{\left(y_{i-1}-y_{i+1}\right)}^{2}} \end{array}$
- Step Width: $w_{i}=\frac{|k_{i-1}x_{i}-y_{i}+b_{i-1}|}{\sqrt{1+k_{i-1}^{2}}}$
- Step Angle: $\theta_{i}=tan^{-1}\left(w_{i}/l_{i}\right)$
- Stride Length: $s_{i}=l_{i}+l_{i+1}$

where the angle $\theta_{i}$ is approximated based on the step length and width. The stride length $s_{i}$ is estimated by computing the sum of two adjacent step lengths.

### 3.4. Gait Health Indicator Extraction

The gait health indicators we extract include cadence/walking speed, left–right symmetry, gait balance, and initial contact type, which reflect different aspects of the gait. In this section, we formulate quantitative scales for these indicators using floor vibration signals and discuss the physical insights behind the formulation.

#### 3.4.1. Cadence/Walking Speed Estimation

The cadence/step frequency is estimated by counting the number of footsteps per 10 s (i.e., $n_{10}$) based on peak-picking on the sum of wavelet coefficients around the natural frequency range of the floor. For example, Figure 9 (right) shows that there are $n_{10}=12$ peaks (i.e., footsteps) within the 10-s window, which means the step frequency is $f=\frac{n_{10}}{10}=1.2$ steps/s and the cadence is $c=6n_{10}=72$ steps/min. The measurement is only related to the temporal aspect of the gait.

The walking speed, on the other hand, is related to both spatial and temporal information. In our approach, the walking speed $v_{i}$ is the step length $l_{i}$ divided by the step time $t_{i+1}^{s}-t_{i}^{s}$, estimated as

(6) $$v_{i}=\frac{l_{i}}{t_{i+1}^{s}-t_{i}^{s}}$$

For example, if a person has a step length of 0.5 m and a step time of 0.5 s, then the walking speed at that step is calculated as 1 m/s.

#### 3.4.2. Left–Right Symmetry Estimation

In this study, we focus on the left–right symmetry during the stance time. This is because the stance time is when the foot contacts the floor, which is directly associated with the force transmission through the body, manifesting the left–right weight distribution. We consider three aspects when assessing symmetry, including the (1) temporal, (2) spatial, and (3) kinetic (force) measurements of the left and right foot. These correspond to the stance time, step length, and the signal energy normalized by the exponential of step-to-sensor distance.

With the above measurements, we describe symmetry using the absolute symmetry index (SI), as introduced in Section 2.2.3. This is because it does not require the classification of the left and right foot and focuses on the absolute difference between the two feet. The SI is defined as below:

(7) $$SI=\frac{2|X_{R}-X_{L}|}{X_{R}+X_{L}}$$

where $X_{L}$ and $X_{R}$ refer to the measurements of the left and right foot. In our approach, the stance time and the step length are used for temporal and spatial SI. The kinetic measurement (i.e., the ground reaction force) is represented by the normalized signal energy. This is because our prior work found that the ground reaction force can be estimated through the signal energy compensated by the wave attenuation effect, which depends on the distance between the footstep and sensor locations [42].

#### 3.4.3. Gait Balance Quantification

Our approach describes gait balance based on the variability of walking to enable balance assessments in more realistic, natural walking settings than clinical gait analysis. Similar to the symmetry measurement, we consider three aspects when assessing balance, including (1) temporal, (2) spatial, and (3) kinetic (force) measurements for balance, which correspond to step time, step width, and the signal energy normalized by the exponential of the step-to-sensor distance, respectively.

With the above measurements, we quantify gait balance using a balance score (BS), as discussed in Section 2.2.3. The BS is defined by accumulating the difference between an individual footstep and the mean of all footsteps within the same trace, computed as follows:

(8) $$BS=\sqrt{\sum_{i=1}^{N}\frac{1}{N}{\left(\frac{X_{i}-\bar{X}}{\bar{X}}\right)}^{2}}$$

where $X_{i}$ is the measurement of an individual footstep, $\bar{X}$ is the mean measurement of all footsteps within a trace, and *N* is the number of footsteps in that trace. *X* corresponds to step time, step width, and normalized signal energy, respectively.

#### 3.4.4. Initial Contact Type Prediction

The initial contact type is predicted by a machine learning classifier using frequency domain features discussed in Section 2.2.3. First, we take the wavelet coefficients from the wavelet decomposition in Section 3.2 to compute the coefficient sum over the frequency axis. Then, we divide the frequency axis into 10 Hz frequency bins and compute the mean of each bin as features to represent different types of contacts. Next, we train a Support Vector Machine model with a Gaussian kernel to capture the non-linear dependencies among various frequency components and predict the initial contact type. To improve the interpretability of the model predictions, we transform the model confidence score using a softmax function to produce the probability of each class. To this end, the outcome of this data pipeline is the probability of each initial contact type, allowing further decision-making by human experts.

## 4. Evaluation

To evaluate our approach, we conduct real-world experiments with 20 participants walking on concrete and wooden floors. In this section, we first introduce the experiment setup and then discuss the results. Furthermore, we design diagrams to visualize the personal gait profiles that enables detection of gait abnormalities.

### 4.1. Real-World Experiment Setup

**Figure 13:**
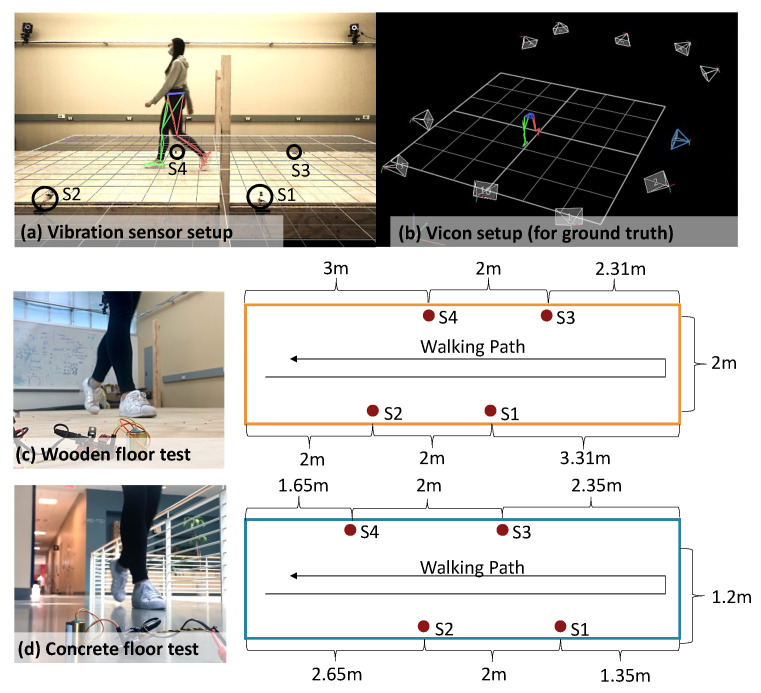
Experiment setup for (**a**) four vibration sensors mounted at the edge of the walkway [36], represented as S1, S2, S3, and S4. (**b**) a Vicon Motion Capture system with lower body locomotion for ground truth collection [43], (**c**) wooden floor test layout, and (**d**) concrete floor test layout.

The experiment involves two sets of sensors: (1) eight SM-24 geophone sensors (produced by Input/Output, Inc. at Stafford, TX, USA) mounted on the surface of the floor for vibration data collection and (2) a Vicon Motion Capture (MoCap) system (produced by Vicon Motion Systems Ltd at Centennial, CO, USA) with 10 infrared cameras to record the ground truth of body movements during gait cycles [43]. The experiment is conducted in a large lab space consisting of two floor types that are commonly used for residential construction: (1) a mounted wooden structure with a wooden surface; (2) a concrete walkway, which is part of the existing building. The MoCap cameras are mounted on the steel bars around this lab space, with adjustable shooting angles and movable tripods to adjust their locations. For each floor type, we installed four sensors at the side of the walking path, spaced 2 m apart. Figure 13 shows the sample experiment setups for wood and concrete floors. The sampling frequency is set to 25.6 kHz to maximize the temporal resolution of the vibration signals for research purposes. A lower sampling rate (around 5000 Hz) is typically sufficient for practical usages.

The experiment involves 20 participants (aged from 18 to 40 years old) walking across one or two types of walkways using their normal gait, and each is repeated for 30 trials back and forth. During each walking trial, 16 markers are attached to the subject’s lower limbs, producing (x, y, z) coordinates of locomotion. The gait events are manually labeled, which include the “foot strike” and “foot off” time for each gait cycle. A total of 12,231 gait cycles are collected and labeled with the ground truth from the MoCap system.

### 4.2. Results and Discussion

Overall, our approach achieved an average of 90.5% (RMSE 0.08 s), 71.3% (RMSE 0.38 m), and 92.3% (RMSPE 7.7%) accuracy in estimating temporal and spatial gait parameters, and gait health indicators, respectively. The accuracy is computed based on the percentage error rate over the absolute ground truth values to describe the overall performance. In this section, we demonstrate and discuss the performance in these three categories and then show the gait profile from all testing participants to visualize the individual differences in gait patterns.

#### 4.2.1. Temporal Parameter Estimation Accuracy

**Figure 14:**
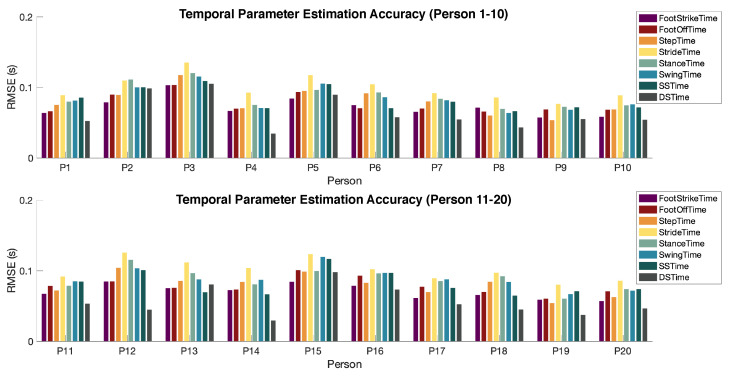
Temporal gait parameter estimation error (RMSE) for 20 participants.

For temporal parameter estimation, our approach has an average of 0.08 s root-mean-square error (RMSE) among all participants. Figure 14 shows the detailed error rate for each parameter per person. The performance of our approach is comparable to the state-of-the-art approaches such as cameras, force plates, and wearables, which have error rates ranging from 5% to 20%, as reported in previous studies [44,45,46]. Overall, the estimation errors are relatively consistent among all participants. Persons 3, 12, and 15 have slightly larger errors compared to the rest of the participants. This is because they have larger variations in temporal parameter values, leading to a less-accurate estimation of dominant frequency ranges.

The error distribution among various types of parameters is also consistent across all subjects. In particular, stride time has the largest error due to the error accumulation in step time estimations. Double support time has the lowest error because it has the shortest duration among all (typically around 0.2 s). When we compare the RMS percentage error, the double support time has the largest error rate (around 20%) while the step time and stride time have the lowest error rate (around 7%).

#### 4.2.2. Spatial Parameter Estimation Accuracy

For spatial parameter estimation, our approach has an average of 0.38 m length (RMSE) among all participants, which means a 28.7% error rate when compared to the absolute value of spatial parameters. Compared to the previous study, our system achieved a 3× error reduction in footstep localization [33].

**Figure 15:**
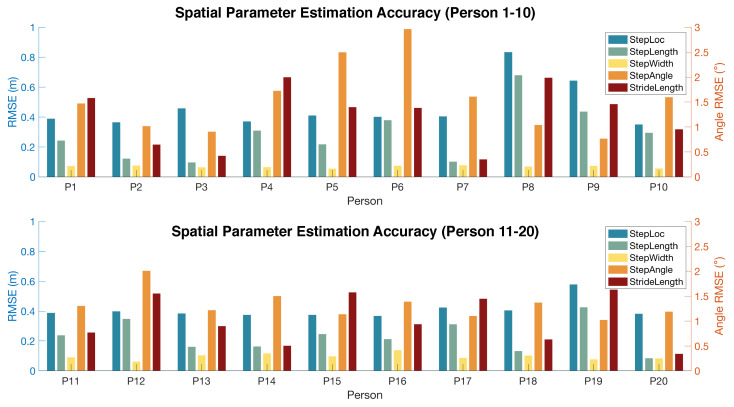
Spatial gait parameter estimation error (RMSE) for 20 participants.

Figure 15 shows the detailed error rate for each spatial parameter per person. Similar to the temporal parameter estimation, the errors for spatial parameters are also relatively consistent among all participants. We observe that persons 8, 9, and 19 have slightly larger errors than the rest of the participants. This can result from the softer shoes they wear during the experiment which produce less-impulsive signals during the initial contact, making it difficult to detect the exact time of wave arrival.

It is surprising to observe that footstep localization has a significantly larger error than the step length estimation. This may be because the localization error tends to bias towards the same direction due to the assumption of the same wave velocity across various directions. Therefore, the bias is mitigated by taking the Euclidean distance between the estimated locations of two adjacent footsteps.

Among the spatial parameters, step width has the lowest RMSE due to its small value (typically around 0.15 m). When comparing the percentage errors, the stride length has the lowest error rate (only around 5% over the stride length value) while the step width has a high error rate (around 18% over the step width value). This is because the stride length is significantly larger than the width and also has less variation within a person. While the step angle only has an RMSE of 1.44 degrees, the error rate of the step angle is high because all the participants walked in a relatively straight line during the experiment, leading to small step angles in all recorded data. To this end, a high error rate does not necessarily mean unsatisfactory performance.

#### 4.2.3. Gait Health Indicator Estimation Accuracy

**Figure 16:**
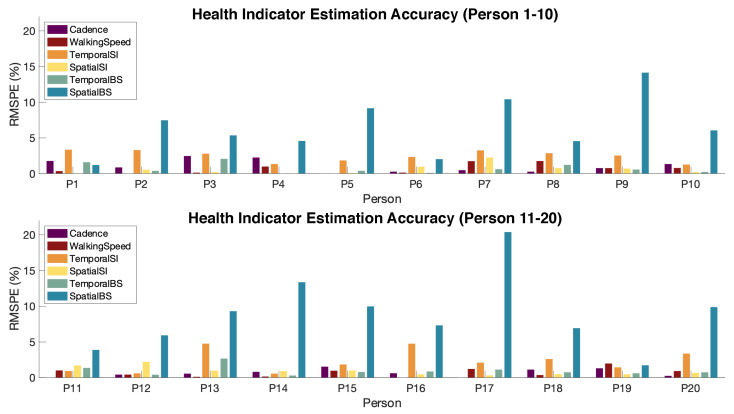
Gait health indicator estimation error rate (RMSPE) for 20 participants.

For gait health indicator extraction, our approach has an average of 7.7% root-mean-square percentage error (RMSPE) among all participants, as shown in Figure 16.

The majority of the errors are less than 5%, except for the spatial BS. This is because the spatial BS is computed based on the estimated step width, which has a large error rate due to its relatively smaller value compared to the spatial resolution. Such error propagates into the BS estimation. In fact, the accuracy of gait health indicators estimation significantly relies on the accuracy of the temporal and spatial parameter estimation. In this evaluation, we did not include the force SI, force BS, and contact type prediction because the ground truth (i.e., force measurement) is not available. According to prior work on vibration-based force and contact type estimation, the evaluation results for the force SI and force BS are 90.2% and 86%, respectively [23,42].

#### 4.2.4. Personalized Gait Profile

**Figure 17:**
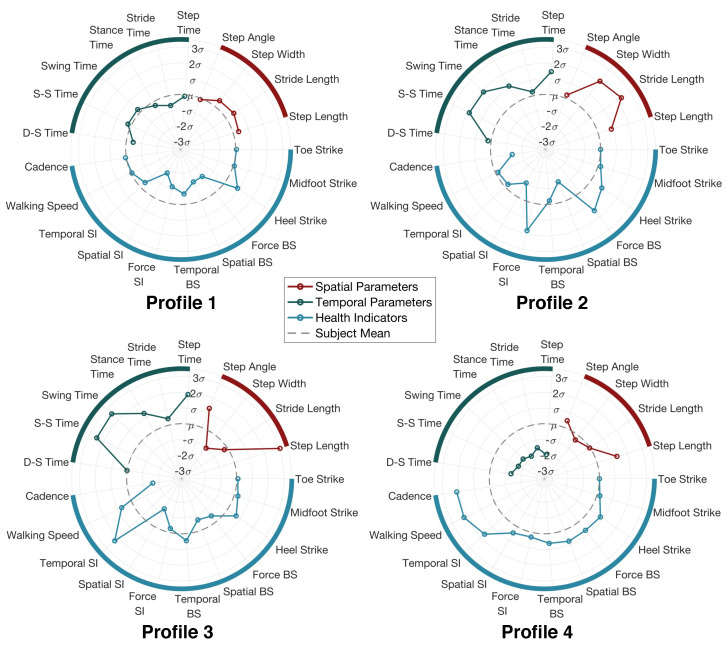
Four typical gait profiles from 20 participants. The axes in each profile represent the estimated temporal gait parameters (green), spatial gait parameters (red), and gait health indicators (blue). The gray dotted line represents the mean value among all participants.

To visualize the gait parameters and gait health indicators among each individual, we summarize all the results above and create personalized gait profiles for all human subjects. A personalized gait profile shows the deviation of each person’s gait from the average gait among all people during the experiment, which provides a direct visualization for the person to understand the style of walking compared to other people. In addition, these profiles can also help with detecting gait abnormalities and tracking rehabilitation stages for patients, which will be explored in our future work. Figure 17 shows the four typical profiles we observed from the participants.

- *Profile 1 “The Steady Walker”:* This person’s gait parameters are all within one standard deviation from the mean value. It means this person has a gait pattern that is close to the average of all walkers during the experiment. The person also has a low score for symmetry and balance, which indicates that the person has good symmetry and stability.
- *Profile 2 “The Wide-Based Walker”:* This person has a significantly larger step width than the rest of the subjects. As a result, the stride length and step time may also increase due to the wide base. On the other hand, the footstep forces are less symmetrical and balanced compared to the other subjects. This may be the root cause of the large step width because a wider base can typically help to maintain balance.
- *Profile 3 “The Large-Step Walker”:* This person has a significantly larger step length and step time than the rest of the subjects. This means that the person takes large steps and, thus, the duration of each step also increases. As a result, the person still has a high walking speed while having a low cadence. Based on our record, this is the tallest person among all subjects, which explains this special gait profile.
- *Profile 4 “The Quick Walker”:* This person has significantly smaller values in all temporal parameters while keeping the spatial parameters around the average. This means that the person takes medium steps but with quick left–right foot alternations. As a result, the person has a high cadence and high walking speed.

We summarize the “subject mean” values of gait parameters from our study and compare them with the values from existing studies from a larger population, shown in Table 1.

As we observe from Table 1, the mean and standard deviation from our dataset are consistent with several previous datasets with larger sample sizes [31,47]. It is worth noting that the subjects in our data have slightly slower walking speeds due to the larger step lengths and longer step time. Therefore, the “subject mean” we used to generate gait profiles may be biased towards a slower walking pattern. Overall, the purpose of showing the “subject mean” is to provide a standard reference for medical practitioners to evaluate an individual’s walking style among the overall population, which is inspired by the standard clinical reports for joint angle assessment, which also use the “subject mean” as a reference.

Meanwhile, it is worth noting that everyone has a unique gait profile due to individual differences. From our record, we observe that the variations among the subjects’ gait profiles can result from a mixture of complex reasons. For example, a person’s height and weight are found to be correlated with the step length and time [48]; a person’s emotional status can affect the step frequency [49]; also, the type of shoes a person is wearing can affect the entire gait profile [50]. In addition, we found that the left–right symmetry is affected by the leg length symmetry: there are three subjects that have asymmetrical left and right leg lengths (differing by around 1 inch), resulting in significantly higher SI and BS. In order to isolate the effect due to individual differences and develop a unified scale for various people, the visualization can be used as a tool to compare the dynamic changes in the same person over time. To this end, additional follow-up experiments with the same group of subjects are needed, which will be explored in future work.

### 4.3. Discussion on the Effect of Human and Environmental Variables

In this subsection, we discuss the effect of human and environmental variables on the performance of our approach, including the effect of floor types, sensor locations, walking paths, and change in walking patterns (e.g., gait abnormalities).

#### 4.3.1. Effect of Floor Types

Since floor structures at residential homes are mainly built with wood or concrete materials, we evaluated our approach on these two types of floors. Our characterization results in Section 2.3 validate the observation that changes in material properties mainly affect the dominant frequency and the wave propagation velocity. In addition, it is worth noting that extra-soft flooring such as carpet tends to absorb the footstep force and significantly reduce the amplitude of floor vibration, which may fall below the sensitivity range of the geophones.

**Figure 18:**
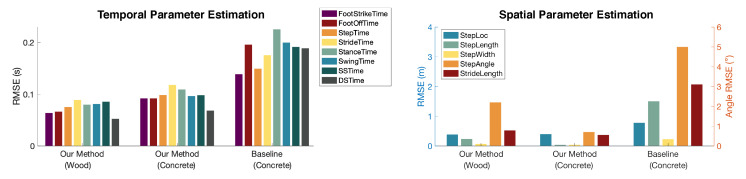
Both spatial and temporal parameter estimation results are consistent across two types of floors. Our system has a significant improvement over the baseline in which there is no floor adaptation when migrating from wooden to concrete floors.

Our approach has consistent results across the two most common floor types (i.e., wood and concrete) based on the data from subjects who walked on both floors, which produces an average of 2.8× and 2.3× error reduction compared to the baseline. As shown in Figure 18, the RMSE of our method on wood and concrete floors is significantly lower than that of the baseline method, despite both following similar trends among various parameters. The baseline method refers to the approach when there is no adaptation to floor types: (1) for temporal parameter estimation, the baseline does not consider the shift in dominant frequency ranges at foot strike and foot off, so it uses the same frequency range for the concrete floor as the wooden floor; (2) for spatial parameter estimation, the baseline does not estimate the velocity profile for the new floor and assumes that the concrete floor has the same velocity profile as the wooden floor. The comparison shows that our approach is robust to various floor types.

#### 4.3.2. Effect of Sensor Locations

The choice of sensor locations is important to achieve optimal performance of our approach system because they affect the data quality and the gait parameters derived from them. First, the distance between the sensors and the footstep is important. In general, the closer the sensors are to the source footstep, the higher signal-to-noise ratio (SNR) the vibration signals will have; however, if the sensors are too close, they may pose trip and fall hazards to the users. So, an ideal distance will be around 1 m away from the walking path. In our evaluation, the four sensors are located in different locations near the walkway, which allows a comparison of the distance effect. Results show that sensors that are closer to the footsteps (within a 2 m distance) have a 5–10% higher accuracy in estimating temporal parameters while having a similar accuracy in estimating spatial parameters. This is because larger signal amplitude leads to more accurate detection of foot strike/off time, while the TDoA approach for spatial estimation requires the wave to propagate for a certain distance in order to acquire sufficient resolution of the time difference.

In addition, the relative location among sensors is important. Based on preliminary testing, we found that placing the sensors on both sides of the walkway has better performance than placing them on a single side, which results in slightly higher accuracy (3–5% reduction in estimation error). This is because placing sensors at both sides captures more spatial information across the floor surface.

Moreover, the number of sensors should be sufficient to cover the area of the floor structure. Although four sensors are used in this evaluation to provide redundancy for sensitivity analysis and discussion, a typical sensing area of 15 m^2^ on the wooden floor or 6 m^2^ on the concrete floor can be covered by one sensor only. The cause of the difference is that the concrete floor is more rigid than the wooden floor, leading to more attenuation in the vibration wave. Our results show that using only one sensor (with an average data quality) still leads to similar accuracy in temporal gait parameter estimation, which means that the number of sensors can be reduced significantly in practice. However, the spatial parameter estimation requires at least three sensors in order to compute the time difference of wave arrival in a 2D plane.

#### 4.3.3. Effect of Walking Paths

Walking path can have a significant impact on the frequency and amplitude of the vibration signals, mainly due to the (1) varying distance between the footstep and the sensor, and (2) the heterogeneous layout of the structural components underneath the floor. The effect of distance between the footstep and the sensor is discussed in Section 4.3.2; so, we focus on the discussion of structural layout in this subsection. As a person walks, each footstep lands at a different location from the previous step; so, the force is applied to a different location where the structural components underneath may change. For example, stepping on a beam typically produces slightly higher frequency and lower amplitude signals than walking in the middle of a floor slab. This is because the beam is typically more rigid and less flexible than the floor slab, and thus results in a slightly higher vibration signal amplitude (∼0.2 V) and higher natural frequency (∼5 Hz) given the same footstep force. Therefore, a different walking path can result in changes in signal patterns, especially when the structural layout is significantly heterogeneous.

To understand the effect of the walking path due to the heterogeneous structural layout, we compare two walking directions (i.e., a person walks from left to right and vice versa in Figure 13) to ensure that the effect of footstep-to-sensor distance is minimized. Both the wooden floor and concrete floor have consistent error rates for these two directions because the footstep locations in both paths cover diverse locations of various structural components. Therefore, slight changes in individual footsteps are not obvious when considering a large group of diverse footstep locations for overall performance. To better understand the cases when the walking path swings from side to side or turns in the middle, additional experiments are needed in the future to cover more areas of the structure.

#### 4.3.4. Effect of Gait Abnormalities

Gait abnormalities are a group of walking patterns that deviate from the normal pattern, such as shuffling, dragging, and left–right asymmetry. These abnormal walking patterns affect the performance of our approach mainly through the varying signal amplitudes and irregular foot–floor contacts. For example, left–right asymmetry causes the signal amplitude to vary when the person is altering the left foot and right foot. This may lead to imprecise wave arrival time estimation because of the low SNR resulting from a significantly lighter footstep. On the other hand, dragging induces additional foot and floor contacts, making it challenging to detect and segment gait cycles and, thus, adding bias to the temporal gait parameter results. In order to design a system that is robust to gait abnormalities, further data collection and additional analysis are required to understand the vibration characteristics induced by each abnormality type. Given the large number of gait abnormality types, we plan to explore disease-oriented data models to tailor to the needs of a specific group of patients.

### 4.4. Comparison with the Existing Sensing Systems

In this subsection, we compare the performance of our approach with the existing sensing systems, including different sensing modalities and various sensor types within floor vibration sensing. The comparison covers both qualitative and quantitative perspectives.

#### 4.4.1. Comparison among Different Sensing Modalities

From a qualitative perspective, our approach meets the user expectations for ubiquitous gait analysis in non-clinical settings, allowing low-cost, non-intrusive, and continuous gait health monitoring in daily life. Overall, floor vibration sensing has the benefits of being contactless, non-disruptive, wide-ranged, and is perceived as more privacy-friendly than cameras and microphones.

For quantitative comparison with the other sensing modalities, our approach has comparable error rates to the state-of-the-art sensing technologies in non-clinical settings. Previous studies have evaluated the accuracy of video cameras, pressure mats, force plates, wearable devices, and Wi-Fi/RF-based devices. These systems are reported to have around 0.01 s to 0.1 s error in temporal parameter estimation and 1 cm to 20 cm error in spatial parameter estimation [51,52,53], which is comparable to our approach (0.08 s and 38 cm mean error). Although our approach is limited in footstep localization due to the floor heterogeneity, it has satisfactory accuracy in temporal parameter estimation with various practical benefits such as low cost (less than USD 50 per sensor), easy installation and maintenance (plug into a power outlet and place on the floor, which is 1% data storage of the videos), large coverage (up to 20 m per sensor, which is 0.01% of sensor density of the pressure mat), and no device-carrying. Therefore, our approach is suitable for non-clinical settings that have fewer restrictions on accuracy requirements while being more convenient and can be operated in a longer term.

#### 4.4.2. Comparison among Various Floor Vibration Sensors

Within floor vibration sensing, existing studies have also utilized acceleration-based, displacement-based, acoustic-based, and velocity-based sensors, which have different sensitive ranges and noise levels depending on the sensing mechanism and configuration. This means that the data quality and the amount of gait information will also vary. For example, typical accelerometers are piezoelectric devices or MEMS (Microelectromechanical Systems), which can be sensitive to temperature or horizontal disturbances in the environment. In addition, displacement meters are typically expensive and require reference points for accurate measurement, which is not practical in measuring footstep-induced floor vibrations. On the other hand, acoustic-based sensors indirectly capture floor vibration through a secondary medium of air or solid, which may impair the signal-to-noise ratio (SNR). It is also noisier and can raise privacy concerns as it is sensitive to people’s conversations as well. We use geophone sensors in this work mainly because their sensitive range aligns well with the frequency range of footstep-induced floor vibrations (10–200 Hz). Moreover, the geophone’s uni-axial, electromagnetic sensing mechanism is less sensitive to environmental variations and lateral disturbances [54]. While this work focuses on geophone sensors, the developed approach is applicable to other vibration-based sensors with proper tuning of system parameters.

When we compare the performance of our approach with the existing work using floor vibration, our system covers the most comprehensive set of gait parameters (5× to 10×) and has the largest subject size (5× to 20×). Since the existing work did not estimate the majority of the gait parameters in our system, only a limited scope of comparison can be made. Specifically, previous work reported that the step time estimation is around 0.05 s and the localization error is 0.42 m [20,24], which are similar to our results of 0.06 s and 0.38 m. This shows that our system has a comparable accuracy to the prior work even when the number of subjects is significantly larger and the test cases (different floor types, walking directions, and sensor locations) are much more complex.

## 5. Future Work

The evaluation results demonstrate promising results of using footstep-induced floor vibrations for extracting gait parameters and gait health information. In the future, we will explore the following topics to further advance the field:

- *Explore disease-related downstream tasks:* We will collect data from patients with a specific type of neurological/musculoskeletal disorder (e.g., Parkinson’s, cerebral palsy, muscular dystrophy) or who have higher risks of falls. By comparing the vibration signals from healthy individuals with those from patients, we can identify differences in gait parameters and use this information to develop algorithms that assist with early diagnosis and continuous tracking of their health conditions. For example, our prior work with muscular dystrophy patients has obtained promising accuracy in disease detection [23].
- *Conduct large-scale field experiments with complex walking scenarios:* We will conduct field experiments in more realistic and less-controlled settings such as homes, hospitals, and public places, which will provide a better understanding of the generalizability of the system and the potential for ubiquitous adoption.
- *Integrate with other sensing technologies:* Our system has the potential to be integrated with other technologies such as wearable sensors, mobile devices, and cameras. Such integration can provide users with a more accurate and comprehensive gait health assessment, capturing force, motion, and muscle activation. In addition, other sources of health records such as a person’s daily habits and medical/injury history can also be fused with the vibration data, which can enable personalized health recommendations and interventions tailored to the individual’s unique needs.

## 6. Conclusions

In this study, we introduce a novel approach for gait analysis using footstep-induced floor vibrations. Compared with existing work using wearables, cameras, and force plates, our approach is low-cost, non-intrusive, and perceived as privacy-friendly, enabling continuous gait health monitoring in people’s daily living spaces. To develop our approach, we systematically characterize the relationships between human gait and floor vibrations. To overcome the influence of floor types on the vibration signals, we analyze the vibrations from different floors and develop features and algorithms that are insensitive to the floor but are sensitive to gait parameters. We evaluate our approach through walking experiments with 20 participants across two common floor types and obtain an average of 90.5% (RMSE 0.08 s), 71.3% (RMSE 0.38 m), and 92.3% (RMSPE 7.7%) accuracy in estimating temporal and spatial parameters, and gait health indicators, respectively. To assist with clinical interpretation, we develop a visualization tool to present an informative summary of a person’s gait pattern. 

## Figures and Tables

**Table 1:** Summary of “subject mean” gait parameters in our study compared with existing studies.

Gait Parameter	Mean (Ours)	Std (Ours)	Mean (Prev. ^1^)	Std (Prev. ^1^)
Walking Speed (m/s)	1.184	0.140	1.267	0.209
Cadence (step/min)	104.1	8.566	114.0	9.300
Step Time (s)	0.581	0.046	0.541	0.041
Stride Time (s)	1.258	0.172	1.090	0.100
Stance Time (s)	0.747	0.066	0.632	0.045
Swing Time (s)	0.415	0.033	0.418	0.025
Single-support Time (s)	0.415	0.033	0.415	0.025
Double-support Time (s)	0.167	0.026	0.133	0.030
Step Length (m)	0.678	0.062	0.613	0.049
Step Width (m)	0.086	0.023	0.091	0.024
Step Angle (∘)	4.123	1.287	4.290	1.800
Stride Length (m)	1.415	0.214	1.398	0.150

^1^ Data recorded from [31,47].

## Data Availability

The de-identified data presented in this study are available on request from the corresponding author.
